# Trouble on the horizon: anticipating biological invasions through futures thinking

**DOI:** 10.1111/brv.13149

**Published:** 2024-09-23

**Authors:** Philip E. Hulme

**Affiliations:** ^1^ Bioprotection Aotearoa, Department of Pest‐Management and Conservation Lincoln University PO Box 85084 Christchurch Canterbury 7648 New Zealand

**Keywords:** anticipatory governance, climate change, crowdsourcing, expert elicitation, forecasting, interdisciplinarity, One Biosecurity, prediction, risk assessment

## Abstract

Anticipating future biosecurity threats to prevent their occurrence is the most cost‐effective strategy to manage invasive alien species. Yet, biological invasions are complex, highly uncertain processes. High uncertainty drives decision‐making away from strategic preventative measures and towards operational outcomes aimed at post‐invasion management. The limited success of preventative measures in curbing biological invasions reflects this short‐term mindset and decision‐makers should instead apply strategic foresight to imagine futures where biosecurity threats are minimised. Here, four major futures thinking tools (environmental scanning, driver‐mapping, horizon scanning, and scenario planning) that describe probable, possible, plausible and preferable futures are assessed in terms of their potential to support both research and policy addressing biological invasions. Environmental scanning involves surveying existing data sources to detect signals of emerging alien species through knowledge of changes in either the likelihood or consequences of biological invasions. Several approaches are widely used for biosecurity including automated scans of digital media, consensus‐based expert scoring, and prediction markets. Automated systems can be poor at detecting weak signals because of the large volume of ‘noise’ they generate while expert scoring relies on prior knowledge and so fails to identify unknown unknowns which is also true of prediction markets that work well for quite specific known risks. Driver‐mapping uses expert consensus to identify the political, economic, societal, technological, legislative, and environmental forces shaping the future and is a critical component of strategic foresight that has rarely been applied to biological invasions. Considerable potential exists to extend this approach to develop system maps to identify where biosecurity interventions may be most effective and to explore driver complexes to determine megatrends shaping the future of biological invasions. Horizon scanning is a systematic outlook of potential threats and future developments to detect weak signals of emerging issues that exist at the margins of current thinking. Applications have been strongly focused on emerging issues related to research and technological challenges relevant to biosecurity and invasion science. However, most of these emerging issues are already well known in current‐day research. Because horizon scanning is based on expert consensus, it needs to embrace a diversity of cultural, gender, and disciplinary diversity more adequately to ensure participants think intuitively and outside of their own subject boundaries. Scenario planning constructs storylines that describe alternative ways the political, economic, social, technological, legislative, and environmental situation might develop in the future. Biological invasion scenario planning has favoured structured approaches such as standardised archetypes and uncertainty matrices, but scope exists to apply more intuitive thinking by using incasting, backcasting, or causal layered analysis. Futures thinking in biological invasions has not engaged with decision‐makers or other stakeholders adequately and thus outcomes have been light on policy and management priorities. To date, strategic foresight addressing biological invasions has applied each approach in isolation. Yet, an integrated approach to futures thinking that involves a diverse set of stakeholders in exploring the probable, possible, plausible, and preferable futures relating to biological invasions is crucial to the delivery of strategic biosecurity foresight at both national and global scales.

## I. INTRODUCTION

Anticipating future threats from alien species to prevent their occurrence is the most cost‐effective strategy to manage biological invasions. Bioeconomic modelling has shown high returns on investment in preventative measures that reduce the probability of biological invasions by animals and plants (Keller, Lodge & Finnoff, 2007; Leung *et al*., 2002; Springborn, Romagosa & Keller, 2011; Epanchin‐Niell, 2017). Yet despite the strong economic incentives for the adoption of effective regulations to support strong biosecurity measures at international borders (Keller & Perrings, 2011; Lodge *et al*., 2016; Leung *et al*., 2014), the evidence points to insufficient proactive action and/or operational tools to stem the tide of invasive alien species worldwide (Ahmed *et al*., 2022; Cuthbert *et al*., 2022). Even in island nations with strict border quarantine regulations, the general trend has been for an increase in the establishment of introduced pests and pathogens over the last decades (Sikes *et al*., 2018; Smith *et al*., 2018). As a consequence, the number of alien species found worldwide is projected to increase strongly by 2050 (Seebens *et al*., 2021*a*).

While the limited success of efforts to prevent biological invasions undoubtedly reflects the limited resources available for investing in biosecurity policies and procedures in many countries worldwide (Early *et al*., 2016) as well as the ever‐growing number of alien species being moved around the world (Seebens *et al*., 2018), a further limitation is the uncertainty regarding the likelihood and consequences of biological invasions. This uncertainty in biological invasions arises not only because of incomplete knowledge but also due to the stochastic nature of the arrival, establishment, spread and subsequent impact of alien species. However, effective prevention requires decision‐makers to be able to forecast both the likelihood and the consequences of an invasion by an alien species before it becomes established in a new region. Uncertainty may drive decision‐making away from investing in preventative measures and towards more tangible outcomes delivered through control efforts implemented after the species has become established and spread (Finnoff *et al*., 2007). Decision‐making under uncertainty and incomplete knowledge is not an issue unique to biological invasions yet there has been limited uptake to date of practical quantitative and qualitative methods developed in other fields to address this problem (Hulme, 2011). Thus, the limited success of preventative measures in curbing biological invasions may reflect a mindset focused on the wrong tools given the level of uncertainty observed. Indeed, it is possible that current attempts to forecast future threats from biological invasions should instead apply strategic foresight and aim to imagine futures where such threats are minimised.

Futures thinking attempts to detect patterns of change, emerging trends, and disruptive events early enough to provide an opportunity for stakeholders to respond and is fundamentally a collaborative process that brings together alternative viewpoints to create multiple narratives of the future (Hines & Bishop, 2007). Strategic foresight encompasses a wide range of tools to support futures thinking and it is important to note that it is not aimed towards better prediction but rather at being better prepared for probable, possible, plausible, and preferable futures (Lustig, 2017). Futures thinking would appear particularly well suited to addressing the problem of biological invasions since the transport, introduction, establishment, spread, and impact of alien species worldwide is characterised by marked spatiotemporal dynamics, a complexity of interacting drivers, uncertainty in outcomes, and has a strong link to human activity and perceptions (Pysek *et al*., 2020). Yet, while process‐based or correlative models of biological invasions are relatively common, the expert‐centred and participatory methods inherent in futures thinking have been undertaken infrequently (Roy *et al*. 2023).

**Fig. 1:**
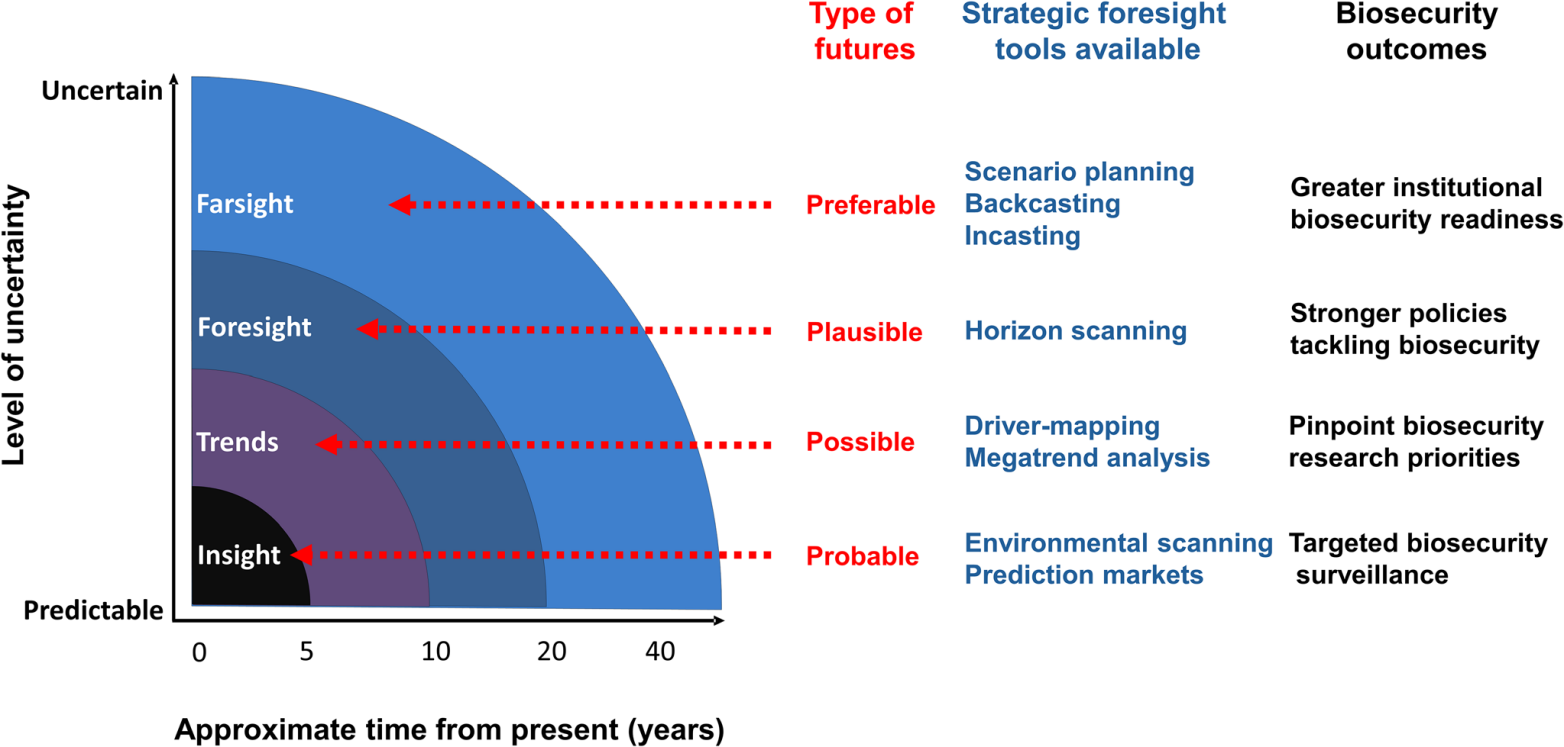
Schematic depiction of the positioning of four different futures along illustrative axes of time and uncertainty. The range of strategic foresight tools described in this review that are best suited for exploring the different futures are also presented. In addition, possible biosecurity outcomes for addressing invasive alien species are described that could arise from using the strategic foresight tools. This graphic extends a simpler diagram described in UK Ministry of Defence (2014).

Despite its application in both government and industry for several decades (De Vito & Taffoni, 2023; Gordon *et al*., 2020), futures thinking has only relatively recently been identified as a potentially powerful tool for biodiversity and conservation planning (Bengston, Kubik & Bishop, 2012; Cook *et al*., 2014*a*,*b*; Sutherland & Woodroof, 2009). A suite of futures thinking approaches exist that can be characterised by how far into the future risks are being explored and the degree of uncertainty this entails (Fig. 1). Thus, there are tools that provide insight by examining a probable future that might occur within the next 5 years (e.g. environmental scanning), through to less certain possible futures derived from surveying likely trends over the next decade and the possible dynamics of change (e.g. driver‐mapping), out to longer timescales that explore plausible futures through foresight (e.g. horizon scanning) and even tackle farsight by investigating what the future might be like to identify preferable futures from a range of alternative options (e.g. scenario planning).

Recent years have seen an increasing trend in the application of different foresight methods to address issues of biological invasions but there remains a strong focus on near‐term insight rather than longer‐term foresight and the current approaches have not received critical appraisal as to their value. Furthermore, while some techniques have been used frequently, several have yet to be applied to address the management of biological invasions. Scope therefore exists to review the potential for futures thinking to support both research and policy addressing biological invasions, highlight gaps and opportunities, and outline an agenda for strategic biosecurity foresight.

## II. ANTICIPATING THE PROBABLE THROUGH ENVIRONMENTAL SCANNING

In relation to biological invasions, environmental scanning involves surveying existing data sources to detect signals of impending alien species threats through knowledge of changes in either the likelihood or consequences of biological invasions. Estimates of the likelihood will encompass a wide range of variables associated with the entry and establishment of an alien species including the number, magnitude, and frequency of introduction pathways, the survivorship and detectability of the alien species along different pathways and the environmental suitability of the recipient environment (Hulme, 2009; Leung *et al*., 2012). Consequences characterise the potential impact of the alien species on social, economic, and/or environmental values that will reflect attributes of the alien species, such as its *per capita* effect and potential geographic distribution as well as characteristics of the recipient environment including the vulnerability to invasion and perceived value (Vilà & Hulme, 2017). Three quite different approaches have been adopted to scan the environment for future biological invasions.

### (1) Scanning multiple information sources to identify future alien pest species

Plant protection organisations have a long history of scanning accessible information drawn from a variety of formal and informal sources to detect new pests, new geographical records of known pests, and new host plants. For example, the European and Mediterranean Plant Protection Organisation (EPPO) has published monthly reports on plant pests since 1951. Today in the era of big data and artificial intelligence, environmental scanning can be automated. The Plant Protection and Quarantine Service of the United States Department of Agriculture launched PestLens in 2014 to coordinate searches for information on target species across nearly 300 scientific journals, more than 50 plant‐health‐related websites, several e‐mail lists related to invasive species issues, and worldwide National Plant Protection Organisation reports (Meissner *et al*., 2015). In 2017, the European Food Safety Authority (EFSA) established an automated system that currently monitors mentions of more than 1000 targeted plant pests from over 12,000 sources in 72 languages from 196 countries, covering all the world's regions (Mannino *et al*., 2021). Although searches are automated, the data retrieved still require processing by full‐time analysts with relevant expertise in pest risk analysis and plant protection.

The promise of automated systems scanning a wide range of digital information sources and feeding information into artificial intelligence algorithms that screen the data to present details of emerging risks has been recognised for well over a decade (Cook *et al*., 2014*a*). The reality is, despite the promise, these systems can be poor at detecting weak signals because of the large volume of ‘noise’ they generate. For example, the PREDICT component of the United States Agency for International Development (USAID) Emerging Pandemic Threats programme was focused on building a global early warning system for emerging zoonotic diseases through internet surveillance of reports of unusual events in hotspot countries, analyses of the capacity of pathogen emergence in hotspots, and sampling of wildlife hosts (Morse *et al*., 2012). Yet the programme was not successful at providing early warning of zoonotic viruses and after a decade was closed down a few months prior to the emergence of the SARS‐CoV‐2 pandemic (Carlson, 2020). In a similar vein, previous information systems that scanned the internet, such as the Australian Biosecurity Intelligence Network and the International Biosecurity Intelligence System, have become obsolete because of their high cost and low success rate (Caley & Cassey, 2023).

### (2) Consensus‐based expert scans to prioritise alien species

Because there is likely to be a temporal lag between the occurrence of an emerging alien species and its mention in social media or scientific publications (Genovesi *et al*., 2024), environmental scanning is not foolproof and there is sense in developing alert lists before species are knocking at the door. Rather than scrutinising recent media, an alternative approach to environmental scanning has been to identify those species most likely to be introduced from the known global pool of potential alien species. In most cases, the pool of potential targets is derived from databases of species known to be invasive elsewhere in the world. Subsets of species identified as having a high likelihood of establishment in a target region have then been ranked using unsupervised machine learning algorithms to detect clusters of likely candidate species (Worner *et al*., 2015). However, an increasing number of studies are applying consensus‐based environmental scanning to anticipate alien species with a high likelihood of entry into a particular region or ecosystem. Although incorrectly describing their activities as horizon scanning, researchers undertaking environmental scans have applied similar consensus‐based approaches to generate alert lists of alien species in the USA (Lieurance *et al*., 2023), Europe (Cano‐Barbacil *et al*., 2023; Lucy *et al*., 2020; Roy *et al*., 2014) and Africa (Kenis *et al*., 2022; Mulema *et al*., 2022). These approaches often generate long lists of potential species that need to be subsequently filtered to produce a ranked list of priority species through a consensus approach that weighs up both the likelihood and consequences of invasion for each candidate species.

### (3) Prediction markets for crowdsourcing specific forecasts of biosecurity risks

Prediction markets allow people to trade contracts that pay based on the outcomes of unknown future events and the market prices generated from these contracts can provide a collective prediction of the likelihood of an outcome (Tetlock & Gardner, 2016). The success of prediction markets in forecasting the results of political elections has led to them being increasingly explored to answer scientific questions. The perceived advantages of prediction markets are that they can bring a wider perspective to bear on forecasting than methods based on expert consensus and provide simple probability estimates that are relatively easy to understand (Thicke, 2017).

To date, biosecurity‐related prediction markets have been used to forecast the likelihood of a human infectious disease outbreak, where they have been shown to provide better forecasts than other methods (Li, Tung & Chang, 2016; Polgreen, Nelson & Neumann, 2007; Tung, Chou & Lin, 2015). Participants are generally pre‐screened prior to invitation to join a prediction market to ensure an appropriate background knowledge and interest in the outcomes being forecast, but the numbers of individuals involved can be much greater than in consensus‐based environmental scanning and the selection is able to draw on a much wider pool of expertise. The potential exists also to provide training to improve the forecasting ability of participants. In contrast to prediction markets in public exchanges where participants stake their own money, those addressing science‐based outcomes provide participants with a set sum of either actual or virtual currency with which to make bets through an online interface. The placing of bets is continuous up until the cut‐off date for any forecast, often just prior to the date of the predicted outcome, thus probabilities of an event are continuously updated and can consider changes in global, regional, or national situations.

The discrete nature of invasive alien species incursions and the short time frame over which forecasts need to be made (e.g. over the next few months or year) make biological invasions amenable to prediction markets. Prediction markets provide individuals with the opportunity to bet on a future biosecurity outcome, such as the arrival of a high‐profile invasive alien species of high concern and for which there is already some familiarity. In contrast to other environmental scanning methods, the outcomes need to be presented in quite specific terms, e.g. will foot and mouth disease establish itself in livestock in New Zealand by 31st December 2025. However, several outcomes can be explored in the same market, either simultaneously or consecutively. The absence of any attempts to use prediction markets in areas other than infectious human diseases suggests that they represent an underexplored opportunity to improve short‐term forecasts of biological invasions. It might be hoped that in the future, standardised designs for prediction markets of biological invasions are established that can make the most of both expert knowledge and artificial intelligence. This would provide the basis for assessing the accuracy of forecasters and prediction markets.

### (4) Recommendations for environmental scanning to address biosecurity threats

Despite the increasing use of environmental scanning to generate lists of potential future invasive alien species, the approach suffers from several limitations.

#### (a) Recognise the challenge of unknown unknowns

The most critical issue is that either the target taxa or the source pool used in environmental scans is generally derived from databases describing species already recognised as being invasive. However, over 50% of all alien species recorded since 1950 have no prior record of establishment in another region (Seebens *et al*., 2021*b*) emphasising that a large pool of species with the potential to become invasive aliens is unlikely to be found in existing databases. This issue likely is less of a problem for identifying invasive alien pests and pathogens affecting human, plant, or animal health where artificial intelligence is being used almost continually to scan open access information to generate alerts of any new threats (Lin *et al*., 2023; Antoniou *et al*., 2024; MacIntyre *et al*., 2023).

#### (b) Establish agreed standards for the relative scoring and prioritising of species

**Fig. 2:**
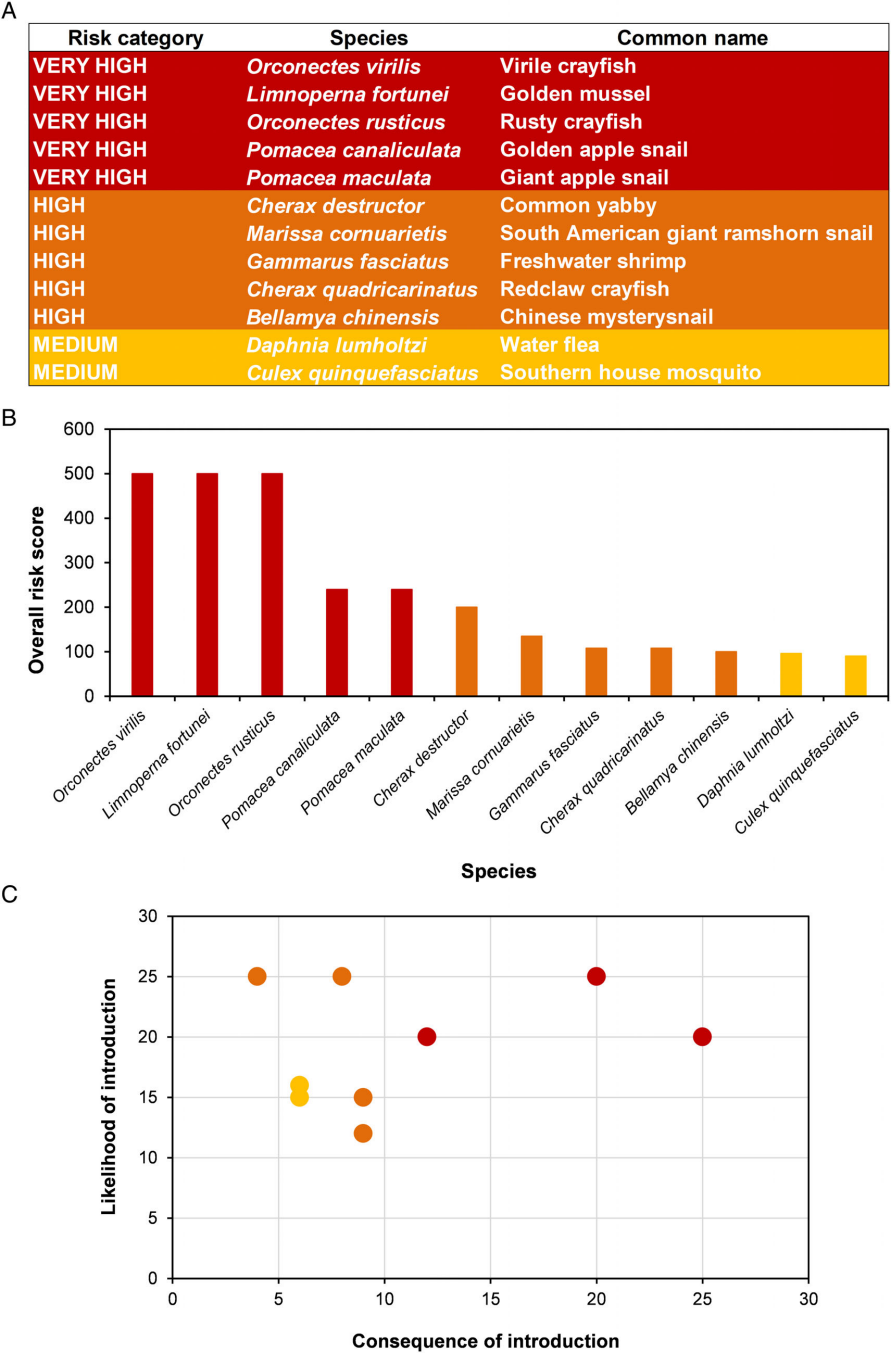
Different ways of presenting results from expert consensus‐based horizon scanning to rank emerging invasive alien species can be misleading. (A) An arbitrary threshold is established to place species in different threat categories, but this can lead to species that pose similar risks being placed in different categories. (B) Presenting the overall scores is preferable but while scores highlight the arbitrariness of broad categorisations, they give no indication of how the scores are derived. (C) Rather than using categories or scores to assess species, the likelihoods and consequences of introduction should be mapped onto a risk matrix that can provide clearer clustering of species in terms of similar risk profiles. Data are from an environmental scan of invasive alien aquatic invertebrates in the Iberian Peninsula (Oficialdegui *et al*., 2023).

Regardless of the complexity of estimating the likelihood of establishment and quantifying consequences on multiple values, expert assessors generally score these components on a semi‐quantitative scale that follows no international standard and thus cannot be compared across different assessments (Lieurance *et al*., 2023; Oficialdegui *et al*., 2023). The application of agreed standards for likelihoods as undertaken by the United Nations Intergovernmental Panel on Climate Change would therefore be valuable (Kause *et al*., 2022). Similarly, there would be benefits of adopting standardised scoring for both environmental (Hawkins *et al*., 2015) and socioeconomic (Bacher *et al*., 2018) consequences of biological invasions. The semi‐quantitative scores are often combined to generate an overall score for which an arbitrary threshold is established to place species in different threat categories (Fig. 2A). Whether scores are combined additively (Oficialdegui *et al*., 2023) or multiplicatively (Lucy *et al*., 2020) can have a major impact on the ranking of species, with a general consensus that the latter is preferable (Tofallis, 2014). However, arbitrary numeric boundaries can lead to species that pose similar risks being placed in different categories (Fig. 2B). Therefore rather than using categories or scores to assess species, the likelihoods and consequences of introductions should be mapped onto a risk matrix, which is a typical feature of risk management standards and guidelines (Duijm, 2015). Plotting likelihoods and consequences of introduction on a risk matrix can provide clearer clustering of species in terms of similar risk profiles (Fig. 2C) as well as highlight biases in the judgement of likelihood and consequences (Smith, Siefert & Drain, 2009). Such an approach helps make the data as transparent as possible so that any cut‐offs in a prioritisation process can be understood and replicated by other researchers, as well as justified to decision‐makers.

#### (c) Address the lack of timely follow‐up action to manage potential threats

Environmental scans can successfully identify short‐range alien species threats such as the quagga mussel (*Dreissena rostriformis bugensis*) being found in the UK 5 months after the publication of an environmental scan that identified it as the species of highest concern (Aldridge, Ho & Froufe, 2014; Roy *et al*., 2014). However, environmental scans of potential invaders are only valuable if they inform decision‐makers in sufficient time regarding actions that should be taken to prevent the entry and establishment of potentially invasive species. Unfortunately, the outcome of most academic analyses tends to be suggestions for further risk assessment or additions to watch lists with little evidence that these are followed up by decision‐makers (Lieurance *et al*., 2023; Peyton *et al*., 2019). Uptake of new information drawn from academic analyses may be limited where government regulators already have established protocols for risk assessment that are embedded in legislation (Jeger *et al*., 2018). Greater emphasis on the options and feasibility of reducing the likelihood and consequences of introducing alien species identified as high priority in environmental scans would be valuable in the future.

Despite the potential benefits of environmental scanning either by consensus or automated searches, it can fail to provide sufficient early warning for any interventions. The red imported fire ant (*Solenopsis invicta*) was reported as a high‐risk threat to Europe through a consensus‐based environmental scan (Roy *et al*., 2019), but by the time these results were published, the fire ant had already become established in Sicily (Genovesi *et al*., 2024). The EFSA environmental scanning for plant health service published its first report of this species becoming established in Europe in 2023, following reports in public media channels (EFSA, 2023) and far too late for the implementation of an effective eradication campaign.

#### (d) Consider using data on near misses

Environmental scanning relies on information from existing databases of accumulated alien species records and/or contemporary reports of changes in alien species distributions or impacts. As yet, the considerable data gathered at the border through quarantine inspections are not widely used to inform future forecasts of risk, regardless of the potential value of such information (Jingjing *et al*., 2023). Detecting temporal trends in the frequency with which certain alien species are intercepted at the border may indicate increasing propagule pressure and a higher likelihood that a species may get through quarantine inspections to establish post‐border.

## III. EXPLORING POSSIBLE FUTURES THROUGH DRIVER‐MAPPING

Environmental scanning provides a means to understand the present threats arising from alien species by analysing information that already exists and thus in itself is rooted in the immediate past (EEA, 2023). Anticipating risk from alien species further into the future requires an understanding of underlying trends that will shape biological invasions over a longer timescale. A key tool in exploring these future trends in the threat of biological invasions is driver‐mapping. Driver‐mapping is used to identify the political, economic, societal, technological, legislative, and environmental drivers shaping the future environment. The terminology describing changes in biodiversity or ecosystem processes distinguishes between direct drivers that unequivocally affect ecosystems (such as climate change, land/sea use change, exploitation of natural resources, pollution, and invasive alien species) and indirect drivers (such as policies and institutions, science and technology, human demography, economics, and societal values) that operate more diffusely by altering one or more direct drivers (Nelson *et al*., 2006). Driver‐mapping aims to explore those direct and indirect drivers that are assumed to be agents of change and systematically examine their context to characterise the most likely future spatio‐temporal trends (United Nations, 2022).

### (1) Limited attempts to map drivers of biological invasions

There has been considerable effort invested in identifying and quantifying the drivers of biological invasions (Hulme *et al*., 2023*b*). A frequent approach has been to examine the strength of correlations between one or more drivers and the number of alien species in a location (Bellard *et al*., 2016; Dawson *et al*., 2017; Pysek *et al*., 2010). However, such comparisons are often limited to the examination of the subset of drivers for which quantitative data exist at national or regional scales. As a result, knowledge of the drivers of biological invasions remains incomplete, emphasises the more tractable drivers over those that require an interdisciplinary approach, and is biased toward studies in developed economies (Hulme, 2022). Under such circumstances, the use of expert‐based consensus to map the magnitude of different drivers on biological invasions is recommended.

Although stakeholder surveys regarding the perceived strength of drivers have been undertaken (Lenzner *et al*., 2020; Paganelli *et al*., 2021) these only provide a static snapshot of opinions and while they may provide input to more deliberative discussions, in themselves they do not constitute driver‐mapping. Attempts at consensus building through expert‐based driver‐mapping for biological invasions have been undertaken infrequently and only at a global scale (Essl *et al*., 2020; Hulme *et al*., 2023*b*). The general procedure has been to identify among the pool of all possible direct and indirect drivers a subset of the most important, and for experts initially to score these drivers in terms of their likely role in increasing the threat of biological invasions. These individual scores are then reviewed using a Delphi approach to expert elicitation to derive a consensus set of scores and associated uncertainties (Hemming *et al*., 2018). Asking participants to assess not only the driver but also its likely temporal trend provides an opportunity to explore different outcomes if temporal trends progress as expected (Essl *et al*., 2020).

**Fig. 3:**
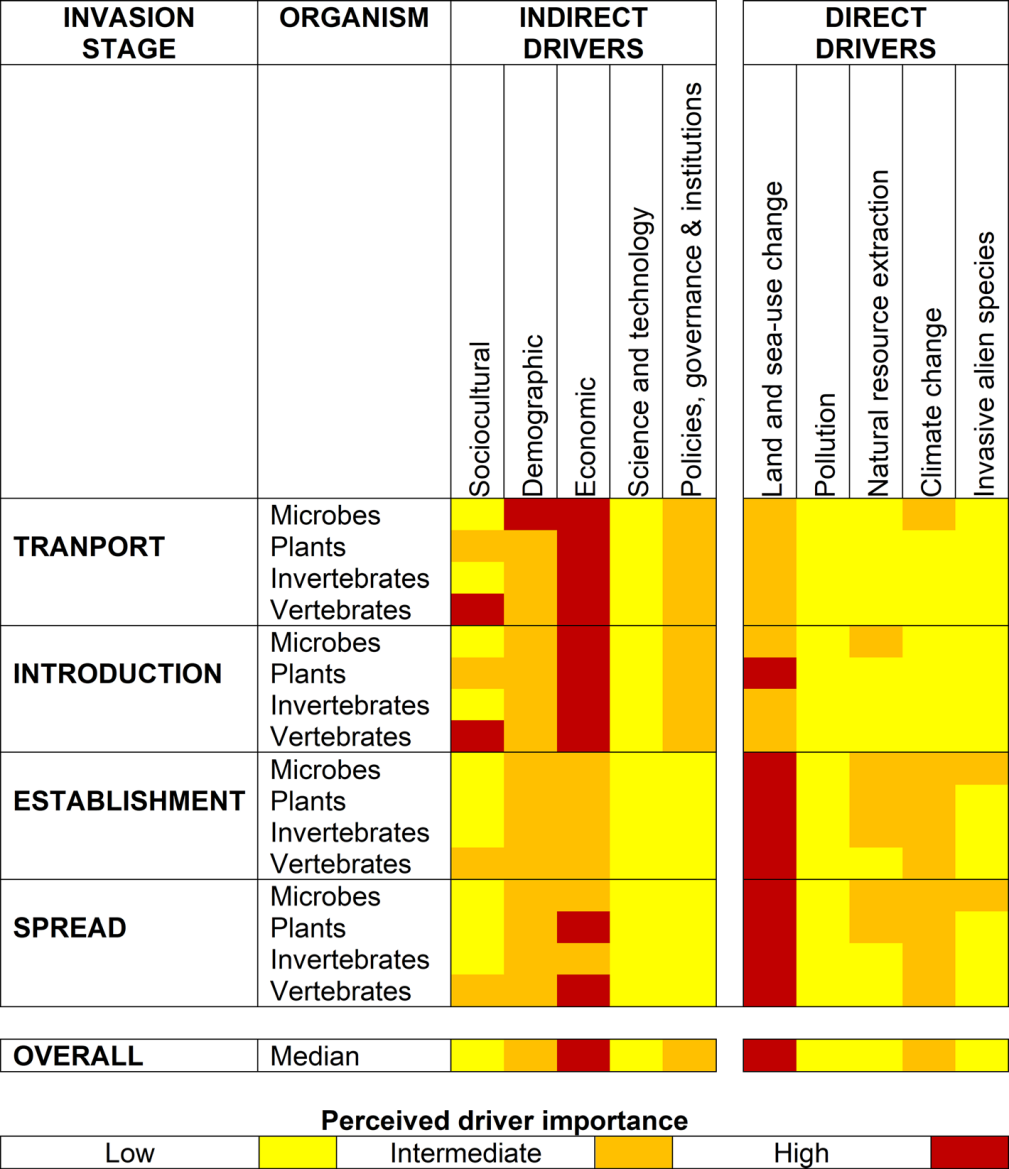
Example of an outcome of driver‐mapping at a global scale for five indirect and five direct drivers of biodiversity change in relation to their perceived importance in the transport, introduction, establishment, and spread of alien microbes, plants, invertebrates, and vertebrates. Data are simplified from an original diagram in Hulme *et al*. (2023*b*).

The few expert‐based assessments to date provide similar insights (Fig. 3). The threat of biological invasions in the future is viewed as being most strongly influenced by the expected increasing trends in global trade and transport, climate change, as well as socio‐economic change and these patterns are consistent for freshwater, marine, and terrestrial ecosystems (Essl *et al*., 2020; Hulme *et al*., 2023*b*). Indirect drivers appear to be more influential in determining the global transport and introduction of alien species while direct drivers generally have their greatest effects on establishment and spread (Hulme *et al*., 2023*b*). The relative importance of different drivers is also expected to differ between emerging and established economies, highlighting that extrapolating perspectives from the Global North to the Global South would be unwise (Essl *et al*., 2020). Driver strength and potential interactions will vary across the world indicating that driver‐mapping needs to be downscaled to examine trends at national or regional scales which also reflect the level at which decision‐making is often undertaken. These insights from previous driver‐mapping are not especially surprising and may simply reflect the state of knowledge regarding drivers rather than an actual measure of importance. This indicates that there is scope to improve driver‐mapping for biological invasions.

### (2) Recommendations for driver‐mapping to address biosecurity threats

There are several ways mapping of biological invasion drivers could be improved.

#### (a) Select the appropriate level of driver detail for subsequent mapping

**Fig. 4:**
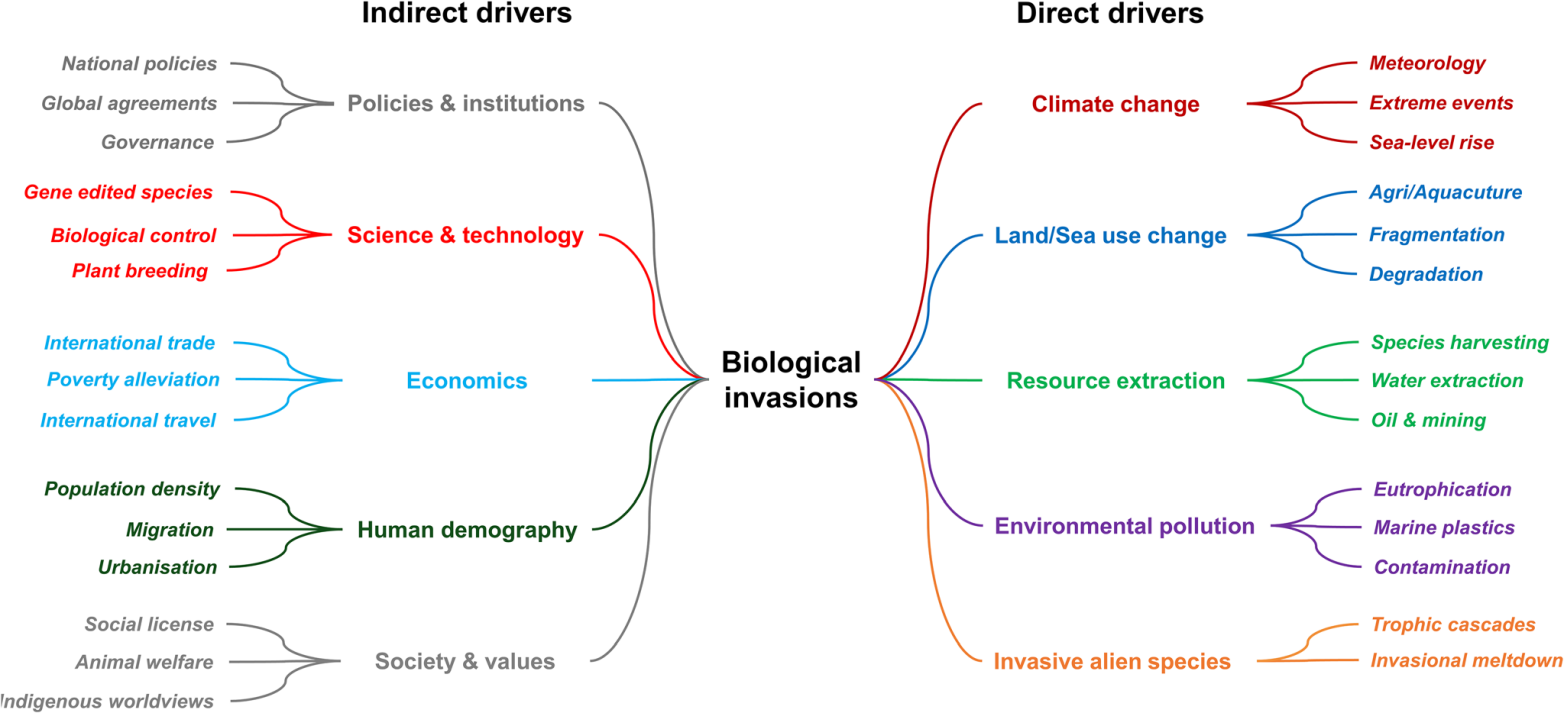
A driver map illustrating the hierarchical nature of main direct and indirect drivers and sub‐drivers of biodiversity change impacting biological invasions. Driver nomenclature adapted from Hulme *et al*. (2023*b*).

Drivers are complex hierarchical processes where each successive lower level includes sub‐drivers or finer distinctions (Fig. 4). Decisions to manage drivers are more likely to be undertaken at the level of sub‐driver (e.g. marine plastics) than at the broader level (e.g. environmental pollution). Relevance trees are a qualitative graphical method that can be used to subdivide the most important drivers into two or more sub‐drivers and examine finer trends that may influence biological invasions in contrary ways as well as find knowledge gaps that could provide direction for further research (Sheppard, 1970). Thus, a hierarchical approach to driver‐mapping is recommended with a first assessment at a coarser level of driver description to identify the subset of drivers that are deemed most important, and then subsequently followed by a finer examination of the specific sub‐drivers among this subset. To facilitate the comparison of driver‐mapping studies, there is potentially a need to establish a globally agreed hierarchical typology for drivers against which driver‐mapping can be undertaken, much like the international standard for introduction pathways (Faulkner *et al*., 2020).

#### (b) Consider examining driver complexes and megatrends

While it is recognised that different drivers interact with each other, the consequences of such interactions are poorly understood for biological invasions (Hulme, 2022; Hulme *et al*., 2023*b*). Creating a consensus‐based system map of drivers affecting biological invasions that identifies both positive (reinforcing) and negative (balancing) feedback loops would help identify leverage points where interventions may be most effective (Matthiessen, Hald & Vigre, 2022). Ultimately, moving research towards quantifying driver complexes may be a useful way forward (Bowler *et al*., 2020). The future trajectory of driver complexes that are likely to have a major impact at a global scale is often captured through the identification of megatrends, which comprise the outcomes of many interrelated trends.

Megatrends are high‐level driving forces already underway that have transformative potential but are nearly impossible to change over the coming decade (Naughtin *et al*., 2024). Many organisations attempt to identify megatrends that are relevant to their particular domain, whether at a global (World Economic Forum, 2024; OECD, 2023) or regional (Barry, 2024) scale or with a specific business (PwC, 2022) or charity (Artuso & Guijt, 2020) focus. The process of deriving megatrends involves first identifying the key drivers of change through driver‐mapping, then clustering drivers that may be closely aligned or correlated and finally undertaking Delphi‐like rounds of ranking and re‐ranking of these driver complexes in relation to their likelihood and consequences. Despite different sources, recent depictions of global megatrends appear to be similar and highlight risks from climate change (e.g. extreme events), technological disruption (e.g. artificial intelligence disinformation), demographic shifts (e.g. involuntary migration), and social instability (e.g. greater inequality) as driver complexes likely to shape the world over the next decade.

Except for the potential of pandemic human or animal diseases, megatrend analyses rarely focus on the implications for biological invasions. An exception is a megatrend analysis of Australia's biosecurity system that examined the implications and policy responses to megatrends arising from changing agricultural systems, urbanisation, greater international travel, loss of biodiversity, and technological innovation (Simpson & Srinivasan, 2014). These megatrends interact to create a potential cascade of problems. Thus, greater international travel increases the risk of pest introduction while the intensification of agriculture reduces its resilience to pest invasions while consumer preference for fewer pesticide residues in food may curtail the tools available to manage any pest outbreaks. These megatrends are unlikely to be unique to Australia, but other regions of the world may be more exposed to other driver complexes, highlighting the value of exploring megatrends. The extensive investment by business and financial organisations around the world to develop megatrends suggest there exists an untapped opportunity to leverage these insights to explore implications for biological invasions at global and regional scales.

## IV. IDENTIFYING PLAUSIBLE FUTURES THROUGH HORIZON SCANNING

Horizon scanning is the systematic outlook for potential threats, opportunities, and likely future developments to detect weak (or early) signals that may evolve into emerging issues that are not yet present in current‐day research or only exist at the margins of current thinking (Cuhls, 2020; EEA, 2023; United Nations, 2022).

### (1) The term ‘horizon scanning’ is often misapplied in biological invasions

Despite a clear definition of horizon scanning, many studies examining biological invasions confuse the longer‐term perspective of horizon scanning with the near‐term information gathered through environmental scanning (Cano‐Barbacil *et al*., 2023; Dawson *et al*., 2023; Oficialdegui *et al*., 2023; Antoniou *et al*., 2024; Cottier‐Cook *et al*., 2024). Although both approaches examine future risks using expert‐based consensus, horizon scanning does not attempt to anticipate specific alien species but rather identify emerging issues that are shaped by future changes to the political, economic, societal, technological, legislative, and environmental drivers of biological invasions. As such, horizon scanning is most effective following systematic driver‐mapping or megatrend analysis that provides a suitable context for detecting weak signals.

Stakeholder surveys of hot topics or priority issues are not in themselves horizon scanning but rather a process of cataloguing and framing disparate ideas, many of which may be well known (Dehnen‐Schmutz *et al*., 2018; Caffrey *et al*., 2014; Kemp *et al*., 2021; Neve *et al*., 2018). While such catalogues of priority questions are a useful first step, horizon scanning relies on intuition and insight which can feel counterintuitive to those who are more used to evidence‐based thinking. Thus, broad surveys of hot topics or priority questions are more likely to capture current rather than emerging issues. In general it is the conversations among experts regarding the scans rather than the specific information retrieved that is the most important aspect of horizon scanning (Wintle *et al*., 2020).

Several guidance documents provide details of how to undertake a horizon scan (EEA, 2023; United Nations, 2022; Boult *et al*., 2018; UNDP, 2022). These guidance documents recommend that horizon scanning involves a team of 10–20 experts in which each expert provides one or more written ‘scans’ of emerging issues that are poorly known but may emerge within the next 10–20 years. The process involves scanning a specific issue by gathering information from a wide range of sources to identify weak signals and long‐term trends that inform future responses (Bengston, Mauno & Hujala, 2024). The number of scans is more important than the number of scanners and usually horizon scanning aims for around 20 scans to be brought to a workshop. The scans should address whether the issue is interesting or new, its scale as well as timing, and what it might mean for the specific topic in the future. These scans are then anonymously reviewed and ranked against predefined criteria by all other experts. Subsequent discussion of the scans and their ranks is followed by opportunities to merge similar ideas, cluster themes, and finally re‐rank the revised scans to derive a short‐list of emerging issues.

### (2) Examples of horizon scanning for biological invasions

Horizon scanning has been applied to identify future challenges of biological invasions (Ricciardi *et al*., 2017), up‐and‐coming priority science areas (Ricciardi *et al*., 2021), and emerging cross‐sectorial innovations to support biosecurity (Hulme *et al*., 2023*a*). The future challenges in biological invasions that have been identified include the application of genomic modification tools to control alien species, commercial use of alien microbes to facilitate crop production, and the fate of intercontinental trade agreements (Ricciardi *et al*., 2017). Horizon scanning by many of the same researchers also identified four priority research areas and these comprised the need to understand the context dependence of alien species impacts, greater emphasis on quantifying the effects of multiple drivers on biological invasions, new approaches to resolve the taxonomic impediment to the identification of alien species, and interdisciplinary research to support the implementation of more robust global biosecurity interventions (Ricciardi *et al*., 2021). By contrast, an interdisciplinary team of human, animal, plant, and ecosystem health specialists identified four emerging cross‐sectorial innovations that could support biosecurity irrespective of the target taxon or ecosystem. These were the implementation of new surveillance technologies adopting state‐of‐the‐art sensors connected to the Internet of Things, deployable handheld molecular and genomic tracing tools, the incorporation of wellbeing and diverse human values into biosecurity decision‐making, and sophisticated socio‐environmental models and data capture (Hulme *et al*., 2023*a*). While these horizon scanning examples addressed biological invasions at a global scale and across broad taxonomic groups and biomes, the approach can also be used for more specific issues such as identifying the next generation of innovative tools for rodent eradications (Campbell *et al*., 2015) or the future threat of anthrax as a biological weapon (Sabra *et al*., 2023).

### (3) Recommendations for horizon scanning to address biosecurity threats

#### (a) Expert teams should embrace cultural, gender, and disciplinary diversity

Horizon scanning appears under‐utilised in the exploration of future trends in biological invasions. However, there are ways that horizon scanning for biological invasions could be further improved. Horizon scanning studies have faced criticism for a lack of diversity among team members, especially when anticipating issues at a global scale (Zenni *et al*., 2017). It is essential horizon scanning is undertaken by experts that are drawn from a wide range of disciplines, including social and natural sciences, indigenous and other marginalised groups, as well as appropriate decision‐makers.

#### (b) Greater effort required to think intuitively and outside of discipline boundaries

Greater diversity is not simply about ensuring an inclusive approach to invasion science (Nuñez *et al*., 2024); without a breadth of expertise, scanning may focus only on detecting change within fields with which experts are familiar and fail to recognise outlier events because of participant cognitive biases (Delaney, 2015). Providing a clearer context for the horizon scan, which could be based on prior driver‐mapping or megatrend analysis, would encourage experts to think outside their normal area of expertise but also highlight gaps in the expertise of the scanning team. Including automated open‐search tools combined with advanced text analytics could support the manual searches undertaken by experts in the early stages of a horizon scan and reduce the risk of personal biases (Wintle *et al*., 2020). Detecting weak signals at an early stage is difficult and the requirement in academic publications to support findings with published evidence can result in the outcomes of horizon scanning being issues that are already recognised and current rather than emerging and underappreciated. It is therefore important to establish a threshold of information that distinguishes weak signals from those issues that are already well recognised so as to focus on the former. While horizon scanning has been used to identify future challenges, priority science areas, or new innovations relating to biological invasions, the societal responses to these findings or the implementation of mitigation strategies are rarely developed and thus results so far have been more informative for researchers than for decision‐makers.

#### (c) Consider disruptive events (wild cards) that might shape the future

The outcomes of horizon scanning exploring the futures of biological invasions have tended to be based on current evidence and trends and thus are often unsurprising. Yet, horizon scanning should embrace a disruptive dimension that challenges current assumptions about the *status quo* and seeks to identify highly improbable events (Delaney, 2015). Asking participants to provide at least one highly improbable event or topic (often termed a wild card) may encourage creative thinking and familiarise the horizon scanning team with the possibility of discontinuities and rare, high‐impact events. These improbable events can be political, economic, environmental, epidemiological, militaristic, or technological. For example, several events with global repercussions were not widely anticipated in the years before they occurred: the fall of the Berlin wall in 1989 and the break‐up of the Soviet Union; the Indian Ocean tsunami in 2004; the financial crisis and global recession in 2008; the SARS‐CoV‐2 pandemic in 2019; the invasion of Ukraine in 2022; and the widespread integration of artificial intelligence and large language models in everyday life in 2024. In their horizon scan, Ricciardi *et al*. (2017) did not anticipate a global pandemic or the rise of artificial intelligence as challenges to invasion science but did identify military conflict as an issue (although not in Europe). Prospective horizon scans should bear in mind that it is possible that some disruptive events are hiding in plain sight but are currently the domain of disciplines other than environmental science while others may be unanticipated secondary effects of events known to be occurring or recently emerging.

#### (d) Identify likely timescales for the occurrence and response to future events

While the aim of horizon scanning is to identify weak signals that will likely grow in importance over the subsequent 10–20 years, it is particularly useful to provide an indicative timeline of events. For example, it might be valuable to estimate how long it might be before a particular event or threat is widely known or reported in mainstream media. Such information would provide insights as to how long stakeholders have to prepare for such an event, whether it is already too late to do anything about it or if it would be premature to act. Ricciardi *et al*. (2017) identified 14 emerging biotechnological, ecological, and sociopolitical challenges relating to biological invasions that were likely to emerge in a subsequent 20‐year time frame. Ranking these challenges in terms of those that might have a high probability of emerging in the short (e.g. within 5 years), medium (e.g. between 5 and 10 years) and long term (e.g. more than 10 years) would certainly assist decision‐makers to prioritise actions and to accommodate or mitigate any consequences. Consideration of timescales could also influence the prioritisation of issues revealed through horizon scanning. Finally, by acknowledging timescales it would be possible to review the outcomes of horizon scans to assess which issues had risen in prominence over the intervening period (Sutherland *et al*., 2019).

## V. DESCRIBING PREFERABLE FUTURES THROUGH SCENARIO PLANNING

### (1) How to plan for intergenerational futures that are uncertain

Scenario planning constructs storylines (also known as narrative scenarios) that are internally consistent and describe alternative ways the political, economic, social, technological, legislative, and environmental situation might develop in the future. These storylines are not meant to capture a future reality but are a means to represent it through a narrative that aims to identify current options with respect to desirable futures (Durance & Godet, 2010). The fundamental reason for scenario planning is that because the future is uncertain, the optimum way forward is to find a strategy that can perform well across several plausible futures. While other foresight methods such as megatrends and horizon scanning can provide perspectives on alternative futures, they are generally not in story form and thus differ from true scenario planning (Bishop, Hines & Collins, 2007). Indeed, scenario planning should deliver storylines that differ strikingly from each other and thus can best test the robustness of any subsequent strategy. Although scenario planning builds on other foresight methods and in particular driver‐mapping and system analysis, it specifically examines the driving forces to identify those that can shift the future along different trajectories. The Millennium Ecosystem Assessment (MEA) represents possibly the first attempt to incorporate environmental concerns into global scenario planning to stimulate thinking as to how human–environment relationships might unfold over the course of the 21st century (Raskin, 2005). Subsequently, scenario planning has been used frequently to explore plausible futures for biodiversity and ecosystem services (Ferrier *et al*., 2016). By contrast, biological invasions have been rarely considered either as an input or outcome of scenario planning (Carpenter *et al*., 2005; van Velden *et al*., 2023).

### (2) Examples of scenario planning for biological invasions

The earliest published example of scenario planning specifically targeting biological invasions attempted to address what the state of invasion by alien woody plants in South African catchments would be in the first 20–30 years of the current millennium (Chapman, Le Maitre & Richardson, 2001). Storylines were developed using the 2 × 2 scenario planning matrix technique (also known as the double uncertainty method) which is one of the most widely used approaches, sometimes claimed to be the gold standard of scenario planning (Bishop *et al*., 2007). The approach examines combinations of high‐impact and high‐uncertainty drivers of change either individually or as driver complexes and identifies opposite and contrasting outcomes for each. Pairs of independent drivers (or driver complexes) are then mapped onto a 2 × 2 matrix describing four contrasting storylines, with most scenario planning focusing on only the two drivers perceived as most important (Curry & Schultz, 2009). The South African scenario planning contrasted a strong *versus* weak national economy and a strong *versus* weak national regulatory environment to create four storylines, only one of which, termed Garden of Eden, would see any effective management of biological invasions (Chapman *et al*., 2001).

Two decades later, rather than revisiting these four storylines, a further set of narratives for biological invasions in South Africa was produced looking out to 50 years in the future and beyond (Wilson *et al*., 2020). The approach mirrored the generic scenario planning archetypes method that describes four overarching predetermined archetypes – Continued Growth, Collapse, Disciplined Society, and Transformative – to imagine alternative futures (Dator, 2009). Continued Growth is the dominant image of the future where change will still reflect the same fundamental processes occurring today, whereas Collapse imagines the breakdown of society and the fear that what is taken for granted today will not continue. Disciplined Society describes society built around fundamental religious, cultural, or political values that constrain future trajectories, often to avoid the alternative of Collapse. Finally, a Transformative future is one that is novel in multiple ways including political, economic, sociological, technological, legal and environmental. Mapping these to the South African scenario planning generated four archetypes: Collapse of Civilisation (Collapse), New Pangea (Continued Growth), Preserve or Use (Disciplined Society) and Conservation Earth (Transformative). The fate of biological invasions under these four archetypes is self‐evident, with New Pangea, Preserve or Use, and Conservation Earth lying on a continuum from bad to better. However, the authors explore the trajectory of these four storylines by reverse engineering to determine the underpinning drivers and outcomes that might guide current strategies to avoid the worst outcomes of biological invasions (Wilson *et al*., 2020).

Scenario planning has been applied at a global scale in the MEA to describe different development paths for ecosystems by examining four archetypes: the expected future, the worst case, the best case, and a highly different alternative (Transformative storyline). Two of the MEA storylines – Order from Strength (worst case) and Global Orchestration (expected future) – describe the situation where environmental policies must react to a world where economic growth is the primary driver while TechnoGarden (best case) and Adapting Mosaic (Transformative) see the world prioritising environmental concerns over economic growth (Carpenter *et al*., 2005). When exploring the consequences of the scenario planning for biological invasions the storylines reporting reactive environmental policies, as expected, result in the increased introduction, spread, and impact of alien species. However, a critically important message that emerges is that even where environmental policies are proactive, stemming the tide of invaders will only be effective through the development of new technologies (Carpenter *et al*., 2005). Unfortunately, two decades on, the world appears to be most closely following the Global Orchestration storyline and despite opportunities for new technologies to support biosecurity (Hulme *et al*., 2023*a*), there are currently no quick technological fixes to the problem of biological invasions.

The 2 × 2 matrix method has also been employed as the basis for global scenario planning of biological invasions. However, rather than only examining a single pair of drivers, pairwise combinations of six driver complexes (climate change, governance, land‐use change, social norms, technology, and trade) were examined to generate 16 storylines, from which four were selected (Ruderal World, Globalized Corporation Society, Hipster/Techno Society, and Fairy Tale) as being generally representative of the contrasting sets of futures (Roura‐Pascual *et al*., 2021). The decision to focus on only four contrasting storylines to summarise the complex set of 16 different alternative futures results in narratives that map closely to the familiar four archetypes: worst case (Ruderal World), expected future (Globalized Corporation Society), best case (Hipster/Techno Society) and transformative alternative (Fairy Tale). A criticism of this approach is that by reducing storylines down from 16 to only four, the majority of the drivers and trends initially generated are discarded, including many that had been judged to be important and highly uncertain. Nevertheless, these four storylines also share similarities with those set out in the MEA but lack the latter's interpretation of consequences of each storyline for the introduction, establishment, spread, and impact of alien species.

These four global storylines have been downscaled for Europe to generate four continental‐scale storylines: Lost in Europe (drawn from Ruderal World), Big Tech Rules Europe (drawn from Globalized Corporation Society), Green Local Governance (drawn from Fairy Tale), and Technological Panacea (drawn from Hipster/Techno Society) that revealed subtle differences from the global storylines (Pérez‐Granados *et al*., 2024). The challenges to manage biological invasions under these four downscaled storylines were then examined in relation to interventions addressing policy, research, public awareness, and biosecurity (Roura‐Pascual *et al*., 2024). The outcome was also similar to that found for the MEA at a global scale (Carpenter *et al*., 2005), with the effectiveness of interventions varying across storylines, being lower where economic growth was prioritised and relying heavily on future technological solutions for successful management of biological invasions (Roura‐Pascual *et al*., 2024).

In contrast to the use of scenario planning archetypes and the 2 × 2 matrix method, participatory visioning focuses on developing representations among stakeholders of desirable futures in order to identify obstacles and opportunities associated with realising a step change in current practices to achieve a transformative future (Jørgensen, 2013). The approach is increasing in popularity in the environmental sciences with the recent development of the Nature Futures Framework (NFF) that is designed to help visualize multiple pathways towards desirable futures for biodiversity and people (Lundquist *et al*., 2021). Participatory visioning has been used to explore future storylines of biological invasions in South Africa, specifically targeting the desirable outcome of more effective management of invasive alien plants (van Velden *et al*., 2023). Applying the Futures Wheel method, participants first identified initiatives that were not currently mainstream but potentially could be game changers and then described the first‐order impacts should these initiatives come about, before putting forward ideas relating to the longer‐term consequences of these initiatives (Bengston, 2016). Although the Futures Wheel can be extended to examine third‐order consequences and can itself provide the storylines for scenario planning, van Velden *et al*. (2023) then applied a Three Horizon Framework to elaborate on the storylines generated during the Futures Wheel in order to understand what needs to change to enable alternative futures to emerge. Participants identified actions across three time horizons where the first horizon reflects the current undesirable situation, the third is the desired future, while the second is the turbulent transitional phase that describes what needs to change to achieve the desired outcome (Sharpe *et al*., 2016). Unfortunately, the researchers did not describe a final set of internally consistent storylines but instead highlighted the social‐ecological nature of biological invasions with interconnected actions needed in multiple sectors (e.g. financial, cultural, social, technological, governance) and emphasised the requirement for a shift in mindset and societal values regarding invasive alien species (van Velden *et al*., 2023).

### (3) Recommendations for scenario planning to address biosecurity threats

#### (a) Increase the range of scenario planning techniques used

Although a conceptual framework for developing scenarios relating to biological invasions has been proposed it strongly emphasises the importance of process‐based or correlative modelling as opposed to expert‐elicitation (Lenzner *et al*., 2019). Given the few expert‐based approaches adopted to date, there remains considerable scope to advance this area of futures thinking to address biological invasions. Despite there being many different approaches to scenario planning (Fancourt, 2016), the examples from biological invasions draw largely from only two: the four archetypes and the 2 × 2 matrix. This may partly reflect that in the most part the scenario planning described above has been undertaken by environmental scientists, often with quite specific interests in biological invasions, and such individuals are known to favour the more structured, repeatable approaches characterised by standardised archetypes and the 2 × 2 matrix (Bishop *et al*., 2007). Yet there is undoubted scope to broaden the composition of the team undertaking scenario planning for biological invasions and under such circumstances using methods that encourage more intuitive rather than analytical thinking would be valuable.

Methods such as causal layered analysis that encourages the incorporation of different worldviews and deeply held myths might be particularly valuable to ensure inclusion of indigenous perspectives (Inayatullah, 1998). Asking participants to describe their personal beliefs regarding invasive alien species may reveal contrasting worldviews that could include invasive alien species being depicted as external existential threats, inherent consequences of monetarist economies, symbols of colonial oppression, untapped economic opportunities, or potential solutions to environmental crises. Including individuals from across the political spectrum and career stages will broaden discussions beyond monetary values (e.g. the cost of managing this species is not worth the benefit) as well as help limit resistance from the past (e.g. we have always done it this way). Bringing decision‐makers and researchers together can help challenge long‐held perceptions of each other (e.g. scientists as trusted experts rather than simply data providers) or their institutions (e.g. government ministries as open libraries for information sharing rather than impenetrable fortresses of confidentiality).

Incorporating a wide range of perspectives is important but can be logistically challenging and thus an alternative is to adopt an incasting approach. Incasting uses existing narrative scenarios as the basis for scenario planning but despite the numerous detailed storylines already produced by policy experts in multinational organisations (OECD, 2023; Barry, 2024; World Economic Forum, 2024) none have been explored in relation to their consequences for biosecurity and biological invasions. Incasting simply requires participants to divide into small groups and read predetermined storylines that they then use to describe the potential consequences for biological invasions. Using a wide range of methods for scenario planning is important since the nature of the storylines is strongly determined by the methods used (Curry & Schultz, 2009). This may mean that the similarity in many of the storylines described for biological invasions is less to do with a consistent set of future trends and more to do with the application of analogous methodologies.

#### (b) Shift from contextual to normative scenario planning and consider backcasting

To date, biological invasions have largely been examined in the form of contextual scenario planning in which the potential changes in the external policy, economic, social, technological, and ecological environment are explored with reference to outcomes for the prevention and management of biological invasions. Contextual scenario planning asks the question ‘What will happen?’. By contrast, normative scenario planning focuses on describing one or more preferred futures and aims to answer ‘How can a specific outcome be reached?’ (Bishop *et al*., 2007). For biological invasions, the preferred future is zero or a reduced threat from invasive alien species, but it is possible that this might result in different preferred storylines depending upon the social and political acceptability of the biosecurity interventions, the availability of new technologies (including novel pesticides), as well as the appetite of politicians, the public, and industry to accommodate some risk.

Participatory visioning is one such normative method, but normative scenario planning also provides an opportunity to work backwards (backcast) from a desirable future to identify the set of policy and research goals that would be required to reach that future. Backcasting avoids the concern that contextual scenario planning can be too safe in their future storylines and allows even impossibly optimistic futures to be considered since these may still point to possible avenues, including breakthroughs, that would otherwise not be explored (Fancourt, 2016). Backcasting should not only point out the policy, economic, social, and technological factors that need to change to achieve the preferable future but also identify the barriers constraining these changes. Both the factors and the barriers can then be mapped along a timeline and the order of events explored to identify how they might interact with each other (Bishop *et al*., 2007).

#### (c) Ensure storylines are relevant to stakeholders addressing biological invasions

Although there have been several attempts to apply scenario planning to biological invasions, it remains unclear to what extent the emerging storylines have had any impact upon decision‐making. Scenario planning can often fail to influence decision‐makers when the objectives are not sufficiently well articulated or integrated into the scenario planning process (Cook *et al*., 2014*a*). It makes sense that the relevant stakeholders are involved in scenario planning since they may bring a different perspective to judging whether the storylines are credible and internally consistent but will also increase the credibility of scenario planning among the stakeholder community. A shift from speculative scenario planning that contemplates a range of plausible futures towards content‐focused approaches that are concerned with developing and evaluating policy options in the face of uncertain but impactful events would require the embedding of decision‐makers into the scenario planning team (Crawford, 2019). Indeed, this is one way in which transformative storylines can be employed not only to help understand the consequences of an unpredictable and novel future but to also identify options as to how to influence or transform the future itself. The method of wind‐tunnelling may be used to assess the likely usefulness or effectiveness of policies or strategies that might be adopted within the scenario planning in order to consider ways to steer society towards desirable storylines and away from undesirable ones (Glover, Hernandez & Rhydderch, 2016).

The challenge of course is that content‐focused scenario planning can end up being constrained by the geographical or sectorial boundaries set by the stakeholders involved and might result in the production of fewer counterintuitive or ‘wild card’ storylines. One option would be to adopt a hierarchical approach that initially generates speculative storylines that are subsequently explored within a clearer stakeholder‐led context. For example, all the biological invasion storylines produced to date appear to map broadly along an economic growth *versus* environmental protection continuum and thus there is scope to engage with decision‐makers to develop more finely tuned storylines along this continuum that address policy and management priorities.

## VI. AN INTEGRATED VISION FOR STRATEGIC FORESIGHT IN BIOSECURITY

### (1) Embrace a wider range of strategic foresight approaches

The foregoing review highlights that the application of futures thinking to address biological invasions is still in its infancy and considerable potential exists to apply strategic foresight approaches to deliver more effective biosecurity. There is a wide range of futures thinking methods that could be exploited more effectively (Hines & Bishop, 2007; Lustig, 2017; Tetlock & Gardner, 2016). For example, Popper (2008*a*) presents summaries of 33 foresight methods including approaches such as morphological analysis, cross‐impact analysis, genius forecasting, role playing, and science fictioning, that have yet to be explored in relation to reducing the risks of biological invasions. As an example, science fictioning scenario planning would require participants to produce short works of fiction, based on scientific fact, written with the aim of starting a discussion about the drivers and consequences of biological invasions from the viewpoint of fictional characters. The dominance of alien narratives in science fiction (Slusser & Rabkin, 1987) should provide an opportunity to subvert this genre and ground it more clearly in the actual threats posed by invasive alien species to society and the environment.

Even in just the area of scenario planning there are at least 20 different approaches including methods such as divergence mapping (Bishop *et al*., 2007). Divergence mapping is a means for participants to brainstorm vignettes of alternative futures, write these on sticky notes, and then map them in terms of which vignettes are closest to the present and those that are furthest away, allowing lines of causality to be discerned and more complex storylines to emerge (Harman, 1976). Of course, with so many approaches to choose from, the task of strategic foresight becomes more complex, especially where the outcomes might be dependent on the methods used. Careful consideration is required when selecting strategic foresight methods, but there is certainly considerable scope to explore new ways of futures thinking in biosecurity and biological invasions.

### (2) Integrate awareness of probable, possible, plausible, and preferable futures

To date, strategic foresight addressing biological invasions has applied each approach in isolation. There are separate studies that undertake environmental scanning, driver‐mapping, horizon scanning, and scenario planning without any clear linkage between them. This is in contrast to most strategic foresight studies that apply multiple methods to achieve their outcomes (Popper, 2008*b*). An integrated approach that brings together environmental scanning, driver‐mapping, horizon scanning, and scenario planning to generate insights into anticipated futures for biological invasions would appear be to be a crucial next step in the delivery of strategic biosecurity foresight.

**Fig. 5:**
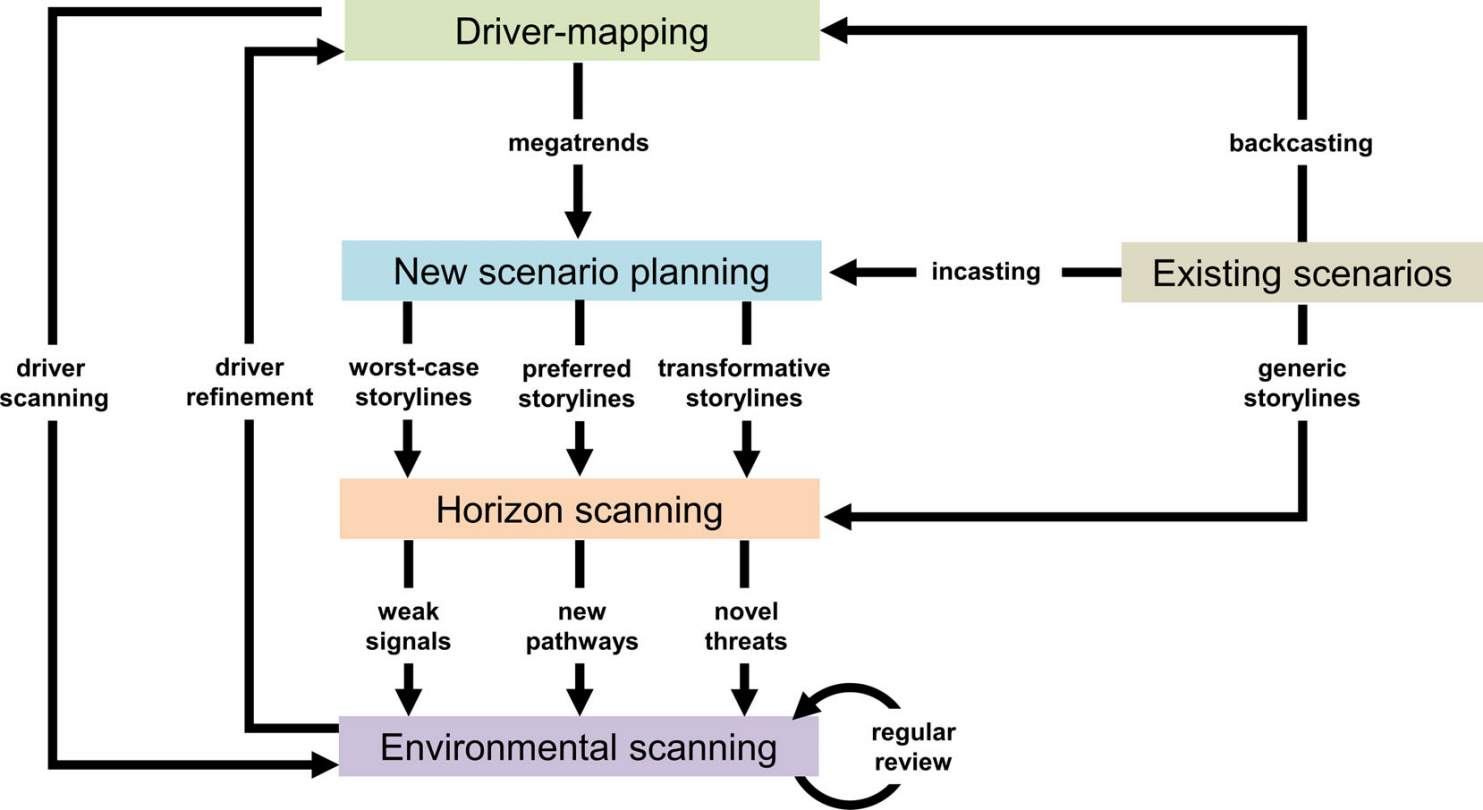
Outline of an integrated strategic foresight approach that links together environmental scanning, driver‐mapping, horizon scanning and scenario planning, illustrating the flows of information and feedback among them. Information gathering is integral to each strategic foresight approach and thus internalised in each box. Depending on the availability of information, decision‐makers and researchers can start anywhere in the diagram. For example, the National Plant Protection Organisations that undertake regular environmental scans could initiate comprehensive driver‐mapping, while policy directorates that have supported previous scenario planning on other topics could use these to kick‐start storylines specific to biological invasions or to inform horizon scanning. A continual review process is inherent in the diagram as new storylines become accepted and come to be existing narrative scenarios allowing for further feedback to driver‐mapping through backcasting.

While it might be tempting to view foresight methods as a series of successive steps along the axes of uncertainty and timescale running from environmental scanning to driver‐mapping then onto horizon scanning and finishing with scenario planning (Fig. 1) a better integrated approach that encompasses feedback between these components may be more effective (Fig. 5). Depending on the availability of information, decision‐makers and researchers can start with any of the strategic foresight methods. For example, the first step for National Plant Protection Organisations that undertake regular environmental scans might be to initiate comprehensive driver‐mapping, while policy directorates that have supported previous scenario planning on other topics could use these to kick‐start storylines specific to biological invasions or to inform horizon scanning.

Nevertheless, a logical starting point for futures thinking is initial driver‐mapping to capture the main driving forces of change and these may be derived by backcasting from pre‐existing narrative scenarios, developed *de novo* through expert consensus, or be evident from recent environmental scans. One of the outputs of driver‐mapping should be broad descriptions of future megatrends that can form the basis for scenario planning. A continual review process is inherent in the procedure as newly developed storylines become accepted by stakeholders and come to be existing narrative scenarios allowing for further feedback to driver‐mapping through backcasting.

Depending on the scenario planning method, storylines may capture business‐as‐usual, worst‐case, preferred, and transformative futures. One or more of these storylines (or storylines from pre‐existing scenario planning) can set the context for horizon scanning to illustrate the emerging issues for research and policy under a particular set of futures. To date, horizon scanning for biological invasions has only examined the business‐as‐usual future but a focus on alternative futures may provide the necessary stimulus for experts to come up with unexpected issues that are completely off the radar. If these issues are classified as occurring over the short, medium, and long term, they can also inform environmental scanning that, rather than only exploring known alien species that are likely to be already knocking at the door, would capture broader issues (e.g. widespread farming of insects as human food) that inform the entire biosecurity system. Rarely has environmental scanning of the future biosecurity threats to specific regions been undertaken more than once over the last decade (Roy *et al*., 2014; Gallardo *et al*., 2016) which is too long a time frame to capture adequately the speed at which political, economic, societal, technological, legislative or environmental drivers change over time. Reviewing environmental scans on at least an annual basis simply to assess whether the original assumptions are still valid or determine if there are reasons to update expectations regarding likelihoods and consequences of alien species arrival as well as the relative strength of drivers and pathways, would appear necessary and should be relatively straightforward.

### (3) Progress strategic biosecurity foresight to deliver anticipatory governance

A fully integrated strategic foresight approach to biological invasions will not be a trivial task that can simply be accomplished by running occasional two‐day workshops and online meetings with an assortment of academic experts. Instead, whether futures thinking for biological invasions is undertaken at a global, regional or national level, a dedicated network of invasion biologists, economists, social scientists as well as decision‐makers will be required to take on this responsibility. Although some personality traits such as cognitive flexibility, and open‐mindedness can help with making forecasts, a key reason for establishing a core network is that participants require training in futures thinking that may include improving inductive reasoning, pattern detection, and creative skills (Tetlock & Gardner, 2016). Thus, over time participants will become increasingly proficient in making robust decisions under uncertainty, confident to challenge their own assumptions, and competent at spotting weak signals of change. However, the same set of individuals should not participate in every step of the integrated approach to ensure a diversity of viewpoints and avoid group thinking that might result in wrong assumptions propagating through the entire process. The teams will also need to avoid the temptation to be too conservative or parochial in their forecasts. Having a core network of forecasters is not unusual, since many organisations, including the European Commission, World Economic Forum, and United Nations Food and Agriculture Organisation, have established their own interdisciplinary foresight networks. However, none of these networks specifically addresses the threat to human health, the economy, and ecosystems from biological invasions and this is another reason why an overarching organisation with responsibility for biosecurity at a global level is warranted (Hulme, 2021).

Yet, even at a national scale, a stronger focus on strategic foresight would be a catalyst for bringing together disparate research and stakeholder communities in human, animal, plant, and ecosystem health to address biosecurity risks (Hulme, 2020). Ensuring futures thinking is integrated and applied in decision‐making requires a shift at the level of institutional processes, infrastructure, operational agility, culture, relationships, and mindsets so that it is embedded in the planning and implementation of policies and programmes (UNDP, 2022). Such an outcome requires anticipatory governance that motivates activities designed to build capacities in strategic foresight by incorporating the perspectives of scientists, policy makers, and other stakeholders to create future plans and execute relevant actions (Heo & Seo, 2021). Despite a strong emphasis on the role of improved governance in the management of biological invasions (Roy *et al*., 2023), there has not been any consideration to date of the importance of anticipatory governance as a means to encourage wider development of foresight systems and to improve pathways to incorporate intelligence about the future into policies addressing invasive alien species. The increasing application of futures thinking in business and government and the availability of a wide range of tools for strategic foresight suggests that the time is right to develop this area more effectively to address the threats from biological invasions.

## VII. CONCLUSIONS

(1) Applying strategic foresight to address the human health, economic, and environmental threats from biological invasions could significantly improve the ability of decision‐makers to undertake preventative action.
(2) To date, most effort has targeted environmental scanning to anticipate the likely entry of known invasive alien species into a particular region, whereas other methods such as driver‐mapping, horizon scanning, and scenario planning have received little attention by comparison.
(3) The heavy reliance by decision‐makers and researchers on environmental scanning for the prevention of biological invasions is unwise due to the high noise to signal ratio of these methods and the difficultly in forecasting alien species with no prior history of invasion.
(4) Rather than target particular species, other strategic foresight tools such as driver‐mapping, horizon scanning, and scenario planning can help assess the robustness of a biosecurity system to identify areas where improvements could be made in response to future political, economic, social, technological, legislative, and environmental changes.
(5) Harnessing the potential of these tools for prevention requires improvements to the approaches adopted to date by invasion scientists, in particular the need to embrace a much more interdisciplinary approach, focus on methods that encourage intuitive thinking and incorporate the perspectives of decision‐makers to ensure that outcomes are relevant to policy and management.
(6) An integrated approach to strategic foresight that brings together environmental scanning, driver‐mapping, horizon scanning and scenario planning in a single overarching framework is the optimum way forward.
